# Design and Simulation of Active Frequency-selective Metasurface for Full-colour Plasmonic Display

**DOI:** 10.1038/s41598-018-29644-8

**Published:** 2018-08-06

**Authors:** Jingjing Guo, Yan Tu, Lanlan Yang, Yin Zhang, Lili Wang, Baoping Wang

**Affiliations:** Joint International Research Laboratory of Information Display and Visualization, School of Electronic Science and Engineering, Southeast University, Nanjing, 210096 China

## Abstract

In this paper, we report a full-colour plasmonic pixel by incorporating a low-index buffer layer and an EO material layer with a gap surface plasmon-based metasuface. The reflection spectra can be modulated by an external voltage bias with a reflectivity higher than 60% when filtering red, green and blue primary light. Vivid colour can be generated by mixing the three primaries in time sequence. Brightness can be tuned by the duty cycle of bright and dark state. Theoretical calculations demonstrate that the switchable pixels we designed can achieve a gamut overlapping 80% area of NTSC colour space and a contrast ratio of 10.63, 26.11 and 2.97 for red, green and blue when using a white quatom-dot-enhancement-film backlit.

## Introduction

Metallic nanostructures for colour filtering have received burgeoning attention due to surface plasmon resonances^1–9^. The resonance is a collective electron oscillation of conduction band in the interface between metal and dielectric. One of the nanostructures is metasurfaces whose optical properties can be flexibly tailored by patterning shape, size and material of compact antenna arrays or slits^10^. Along with the spectral, amplitude and phase tunability, these metasurfaces could realize extreme small pixels for super-high resolution display^11,12^. Although these advantages are truly compelling to replace available technologies, brightness, reflection or transmission efficiency, colour purity and gamut are still struggling in a dilemma. One way is to employ gap surface plasmon (GSP)-based metasurfaces^13,14^, design Febry-Perot cavity into antennas^15^ or utilize Fano interference^16^. Color primaries varied by their structures are encoded into three sub-pixel regions and colours are generated by spatially mixed for the sake of pixel dimensions. To economize space, some researchers incorporate plasmonic surfaces with nematic liquid crystals (LCs) to replace geometry-varied sub-pixels^17–19^. The LCs are used to modulate the polarization state of light, combined with a linear polarizer to filter colours. These colour-changing surfaces increase resolution by at least three times, yet they still meet some problems in low reflection or transmission efficiency and poor colour purity. Other trials, such as gate-oxide metasurfaces, utilize field-effect modulation to realize refractive index variation of conducting oxide^20–24^, whereas the spectral tunability is undesirable. In our previous works, we have demonstrated that electro-optics (EO) material with large EO coefficient could be used to modulate plasmonic colour in the red and green region when being sandwiched between metallic metasurface and metal layer^25^. However, this nanostructure cannot span the entire visible spectra because of an intrinsic losses in short wavelength.

In this paper, we propose a full-colour plasmonic display based on time sequence voltage modulation. This is achieved through low-index buffer materials, high-index EO materials integrated with GSP-based metasurfaces. An active control of resonance shift is realized by electric modulation, leading to red-green-blue (RGB) primaries and even brightness tunability assisted with white backlit. The nanostructure is modeled and analyzed through finite-element-method (FEM). The proposed device exhibits advantages of high reflectivity, narrow bandwidth, flexible colour selectivity and wide gamut. This work is potential for full-color wearable optical applications with super-high resolution.

## Results

### Full-colour plasmonic display

**Figure 1:**
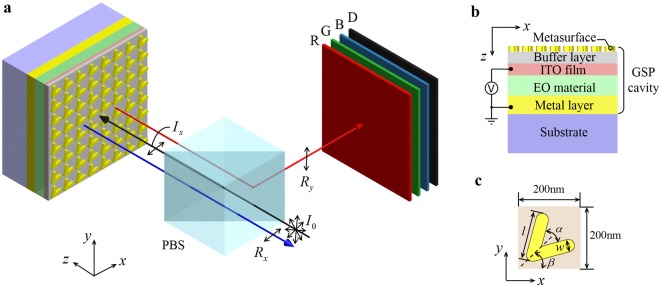
Dynamically tunable full-colour metasurface. (**a**) Scheme of proposed device with white backlit (not shown). Light *I*_0_ passes through a PBS and transfers to *x*-polarized light *I*_*x*_. After being reflected by the GSP cavity, the cross-polarized reflective light *R*_*y*_ carries colour information and is reflected by the PBS, while the co-polarized reflective light *R*_*x*_ transmits through the PBS. (**b**) Cross-section of the GSP cavity consisting of a metasurface, a buffer layer, an ITO film, an EO material layer and a metal reflection layer on a dielectric substrate. The ITO film serves as a transparent electrode while the metal reflection layer acts as a ground electrode. (**c**) A unit cell of the antenna array. The V-shaped antenna has two *l*-long, *w*-wide arms split with an angle *α*. The angle between the symmetric axis and *x*-axis is *β*.

The voltage-induced colours are generated by light scattering from plasmonic nanostructures, as shown in Fig. 1a. At the top of the device, unpolarized light *I*_0_ produced by a white backlit passes through a polarized beam splitter (PBS) and transforms to *x*-polarized light *I*_*x*_. The polarized light continues through an antenna array and some specific frequencies of it excite GSPs in the resonance cavity. Light that is not absorbed by the GSP cavity is out-coupled by the antennas and propagates in the free space. To construct a chromatic plasmonic display, these antennas are grouped into micron-scale arrays known as pixels. Because of these engineered antennas, the out-coupling light *R*_*y*_ is *y*-polarized and eventually reflected by the PBS. The reflection spectra can be tuned by a pulse width modulation (PWM) voltage applied on the ITO film and the metal layer. When the voltage values for reflecting red (R), green (G), blue (B) and dark (D) are obtained, full colour can be display by controlling the duty cycle of them. On contrary, the *x*-polarized light *R*_*x*_ that does not excite GSPs is directly reflected by the metal reflection layer and transmits through the PBS.

Specifically, the GSP cavity deposited on a dielectric substrate comprises a metallic metasurface, a buffer layer, an indium tin oxide (ITO) film, an EO modulation layer and a metal reflection layer as shown in Fig. 1b. Herein, the metasurface is a series of V-shaped antennas of the same geometry, which dominates the polarization conversion in the process of out-coupling. These oriented antennas are arranged in a square array to separate the GSP-selected reflective waves and the direct reflection waves. As shown in Fig. 1c, the unit period is 200 nm both in *x*- and *y*-direction, which is compact enough to function with visible waves. The angle *β* between the antenna symmetric axis and *x*-axis is 45° for a higher polarization conversion efficiency. The arm intersection angle *α*, length *l* and width *w* equals to 60°, 147 nm and 40 nm respectively.

**Figure 2:**
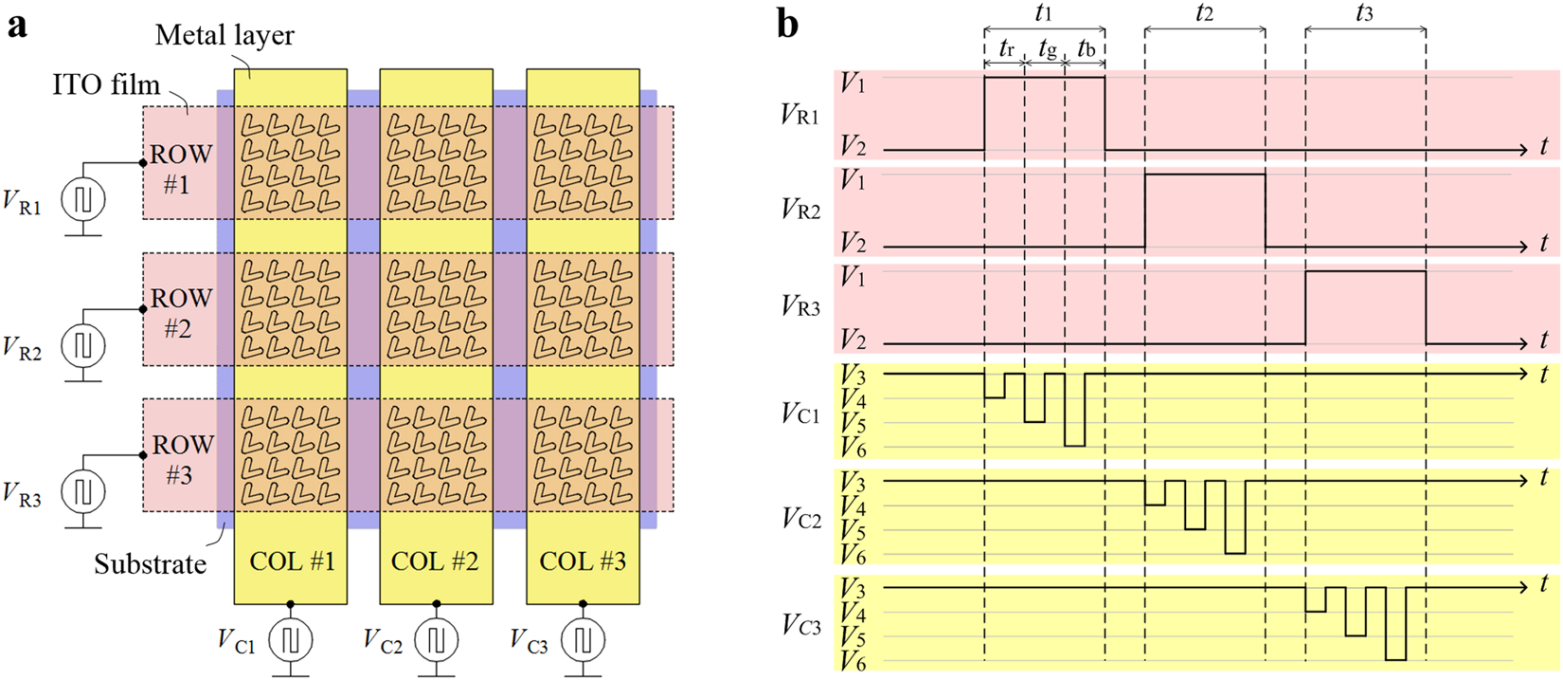
Pixel array scheme. (**a**) 3 × 3 pixel array scheme. *V*_R1_, *V*_R2_ and *V*_R3_ are the voltage values applied on the row electrodes ROW #1, ROW #2 and ROW #3. Similarly, *V*_C1_, *V*_C2_ and *V*_C3_ are the voltage values applied on the column electrodes COL #1, COL #2 and COL #3. (**b**) Time sequence voltage modulation for red, green, blue and dark state, where *V* is voltage and *t* is time. V_*i*_ (*i* = 1, 2, ..., 6) represents the applied voltage values. *t*_1_, *t*_2_ and *t*_3_ is the pulse lasting time for the chosen row. *t*_r_, *t*_g_ and *t*_b_ is the modulation time for red, green and blue. Waveforms in (**b**) are used for modulating colors of the diagonal pixels from the left-top to the right-bottom cross sections in (**a**).

Figure 2a shows the electrode arrangement of the pixel array. The GSP cavities are separated in individual pixels on the substrate. About seven unit periods are needed for close to the resonance wavelength of an infinite periodic structure to a maximal extent^17^. So the pixel size around 1.4μm can be made in our design. The ITO films are assumed as row electrodes while the metal layers are column electrodes, so that the cross sections of them form an *n* × *n* addressable pixel array. The voltage applied to each pixel is the voltage difference between its corresponding row and column. The pixel can be chosen by progressive scanning in a time sequence (*t*_1_, *t*_2_ and *t*_3_ in Fig. 2b): voltage *V*_1_ for colors is applied to the selected row, and other rows are dark at *V*_2_. The brightness of R, G and B can be modulated as follows: the duty cycle of *V*_3_ and *V*_4_ in *t*_r_ controls the brightness of red. Similarly, the brightness of green depends on the duty cycle of *V*_3_ and *V*_5_ in *t*_g_, while that of blue is according to the duty cycle of *V*_3_ and *V*_6_ in *t*_b_. The percentage among *t*_r_, *t*_g_, *t*_b_ in *t*_1_ determines the mixed color.

**Figure 3:**
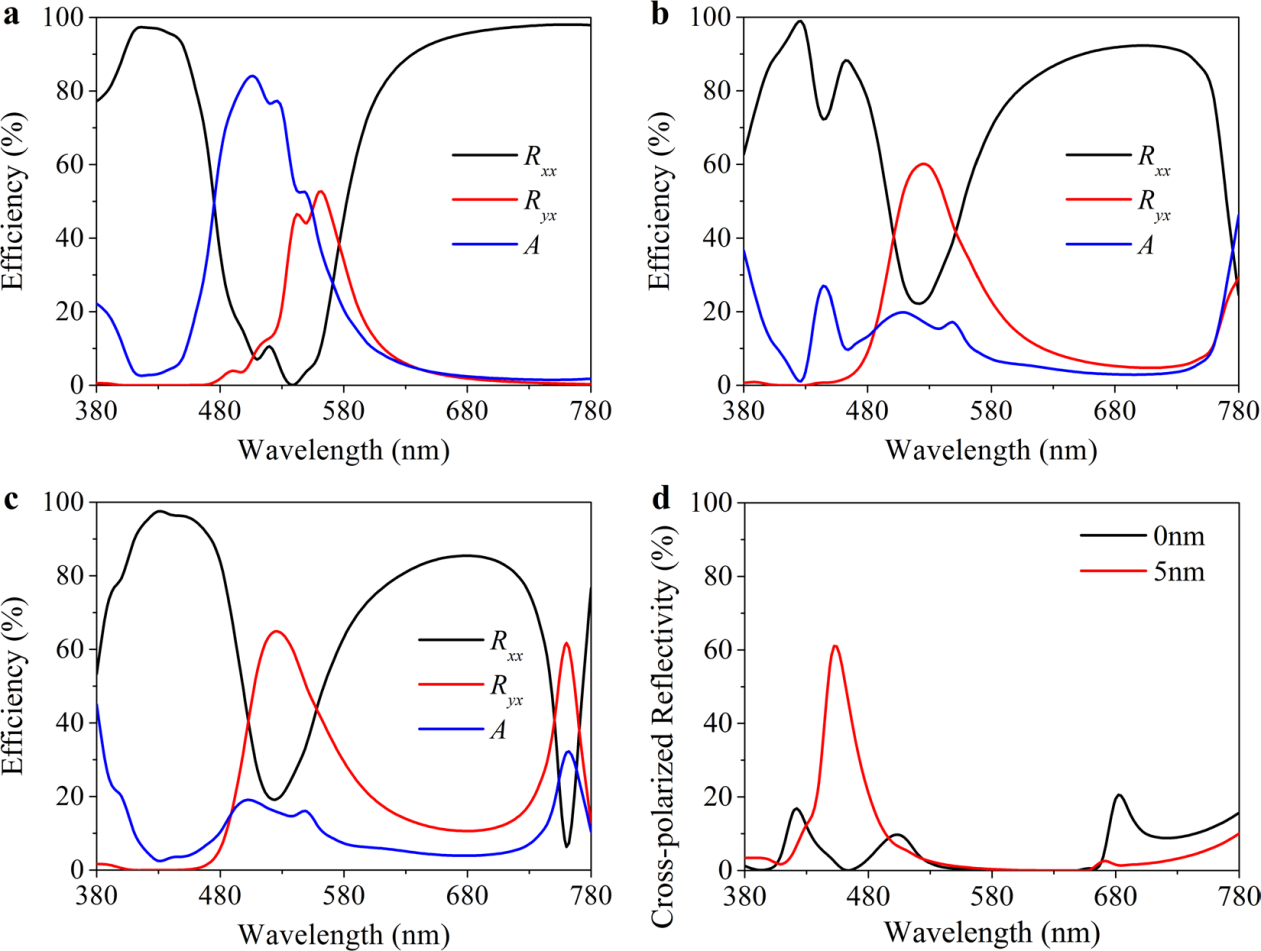
Absorption reduction by the buffer layer. (**a**~**c**) The co-polarized reflectivity (*R*_*xx*_), cross-polarized reflectivity (*R*_*yx*_) and absorption spectra (*A*) at 0 V voltage bias with a buffer layer thickness of 0 nm, 5 nm and 10 nm, respectively. (**d**) The cross-polarized reflection spectra at 25 V with a 5nm-thick buffer layer or without. The thickness of antenna and EO modulation layer is 30 nm and 150 nm respectively.

In our simulation, both the antennas and the reflection layers are chosen as silver. This is because that silver is an ideal metal material for its low cost in the visible regime compared to gold and narrow response spectra compared to aluminum. Additionally, we choose 4-dimethyl-amino-Nmethyl-4-stilbazolium tosylate (DAST) as EO dielectric for the reason that its refractive index can be linearly tuned by external voltage^26^. Also, it can be fabricated as individual crystals blocks^27^. According to our previous research, a typical GSP cavity of metal-dielectric-metal unexpectedly introduces an oscillatory absorption to the reflection light. The loss is particular high in the blue region, confining the working wavelength^25^. The key to realize a full color reflection in this paper is that we use MgF_2_ as a low-index buffer layer sandwiched between the metal antennas and the high-index electric material so as to suppress the loss. This can be possibly explained by the theory of metal cladding dielectric waveguide^28,29^. Figure 3a~c shows simulated spectra at 0 V voltage bias as the buffer layer thickness varies from zero (no buffer layer) to 10 nm. The absorption efficiency declines once the buffer layer is employed. Also, the peak cross-polarized reflectivity gradually rises with the buffer layer thickening, yet an unexpected peak appears in the deep red and infrared region of the cross-polarized reflection spectra. This secondary reflective peak refers to the second order (m = 2) of F-P resonance in Eq. 1. As intensity of white backlit is extremely low in infrared region, the influence of the secondary resonance in Fig. 3b can be eliminated. So a 5nm-thick buffer layer is adopted in our design. When a positive voltage is applied on the ITO film, the resonance wavelength successfully shifts to the blue region for GSP cavity with buffer layer as shown in Fig. 2d.

### Voltage-dependent colour

The optical resonance derived from the standing-waves of GSPs can be characterized by the typical Fabry-Perot resonator formula

1 $$L{k}_{0}{n}_{gsp}+\varphi =m\pi .$$

Here *L* is the width of the nanobrick, *k*_0_ is the vacuum wave number, *n*_gsp_ is the effective refractive index of the GSP, *m* is an integer defining the mode order, and *ϕ* is an additional phase shift. Herein, *n*_gsp_ is depended on the refractive index of metal and dielectric, and the width of the dielectric gap^14^. When the voltage applied on the ITO film shifts, the electric field across the EO modulation layer varies with it, which results in a change on its refractive index. This change determines the effective refractive index of the GSP modes *n*_gsp_ and the additional phase *ϕ*. Hence, the resonance wavelength is altered to satisfy with Eq. 1. Besides, the resonance condition is also depended on the antenna profile, the cavity length and the metal material, yet they are fixed after fabrication.

**Figure 4:**
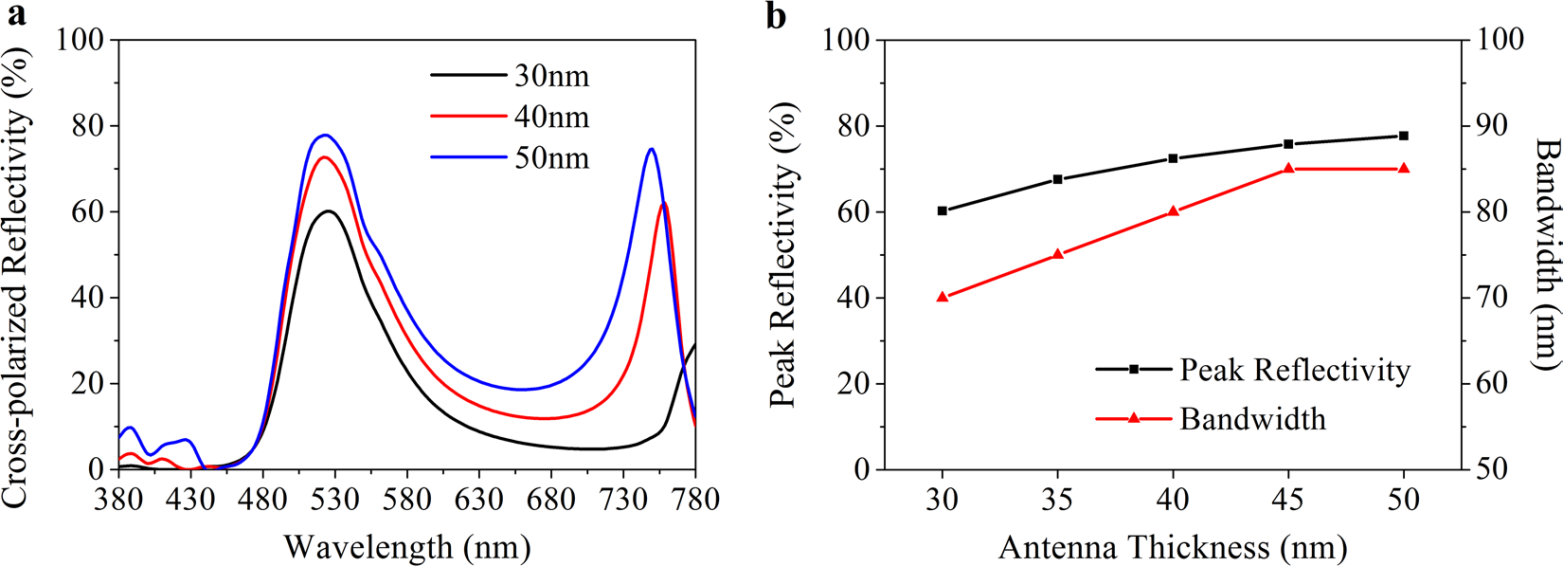
Optical characteristic dependence on the antenna thickness at 0 V. (**a**) Cross-polarized reflection spectra as a function of antenna thickness. (**b**) Peak reflectivity and bandwidth of the spectra in (**a**).

**Figure 5:**
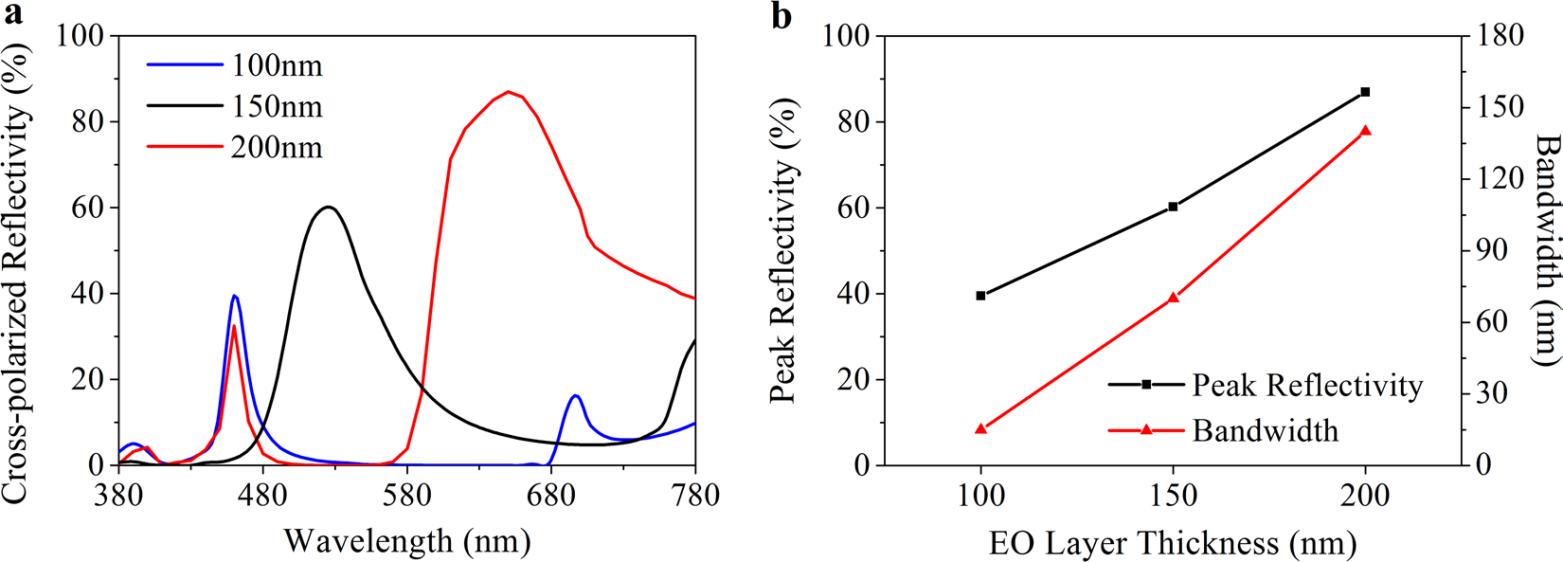
Optical characteristic dependence on the EO layer thickness at 0 V. (**a**) Cross-polarized reflection spectra as a function of EO layer thickness. (**b**) Peak reflectivity and bandwidth of the spectra in (**a**).

**Figure 6:**
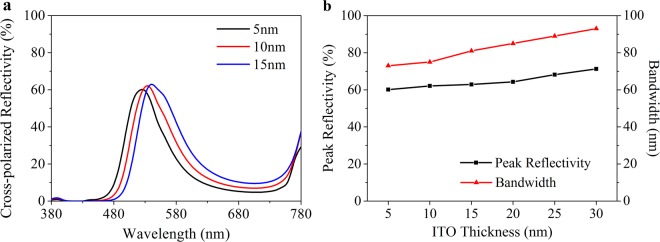
Optical characteristic dependence on the ITO thickness at 0 V. (**a**) Cross-polarized reflection spectra as a function of ITO thickness. (**b**) Peak reflectivity and bandwidth of the spectra in (**a**).

In this paper, a periodic unit cell is modeled to analyze the influence of the antenna, the ITO film and the EO layer thickness on the optical characteristics using finite-element method (FEM), which is described in detail in Method. Wherein the thickness of the metal layer equals to 130 nm, which is referred to ref.^25^. The optical characteristics are firstly analyzed at 0 V. Although the thickness of the antenna has no effect on the resonance wavelength according to the Eq. 1, the thicker one also arouse broader bandwidth and higher reflectivity as shown in Fig. 4. Also, an unexpected secondary reflective peak appears in the deep red region when the antenna is thicker than 40 nm, thereby 30 nm is an optimal choice. Additionally, because the refractive index shift of DAST is linearly related to the ratio between external voltage and its thickness^27^, the EO layer thickness should be as thin as possible for reducing the maximum voltage bias. However, a more compact one companies with a stronger confining GSP mode as shown in Fig. 5, even if assisted with a buffer layer. Also, a blue shift satisfied with Eq. 1 appears with a reflectivity declining and a band broadening in the reflection spectra. So we choose a 150 nm-thick EO layer for further discussion. Another influence factor on cavity length is the ITO thickness. As shown in Fig. 6, the ITO film thickens per 5 nm from 5 nm to 30 nm arouses a 10 nm redshift of resonance wavelength on average. Meanwhile, the peak reflectivity increases from 60% to 71% and the bandwidth widens from 73 nm to 93 nm. For a consideration of better color purity and difficulty of production, we adopt a 10 nm ITO film, which is acceptable for keeping a uniform thickness and electric conduction^30,31^. Therefore, the thickness of antenna array, buffer layer, ITO film, EO layer and metal layer is chosen as 30 nm, 5 nm, 10 nm, 150 nm and 130 nm, respectively. In this condition, our plasmonic nanostructure can achieve relative high efficiency (62%) and narrow resonance band (76 nm) in the cross-polarized reflective spectra.

**Figure 7:**
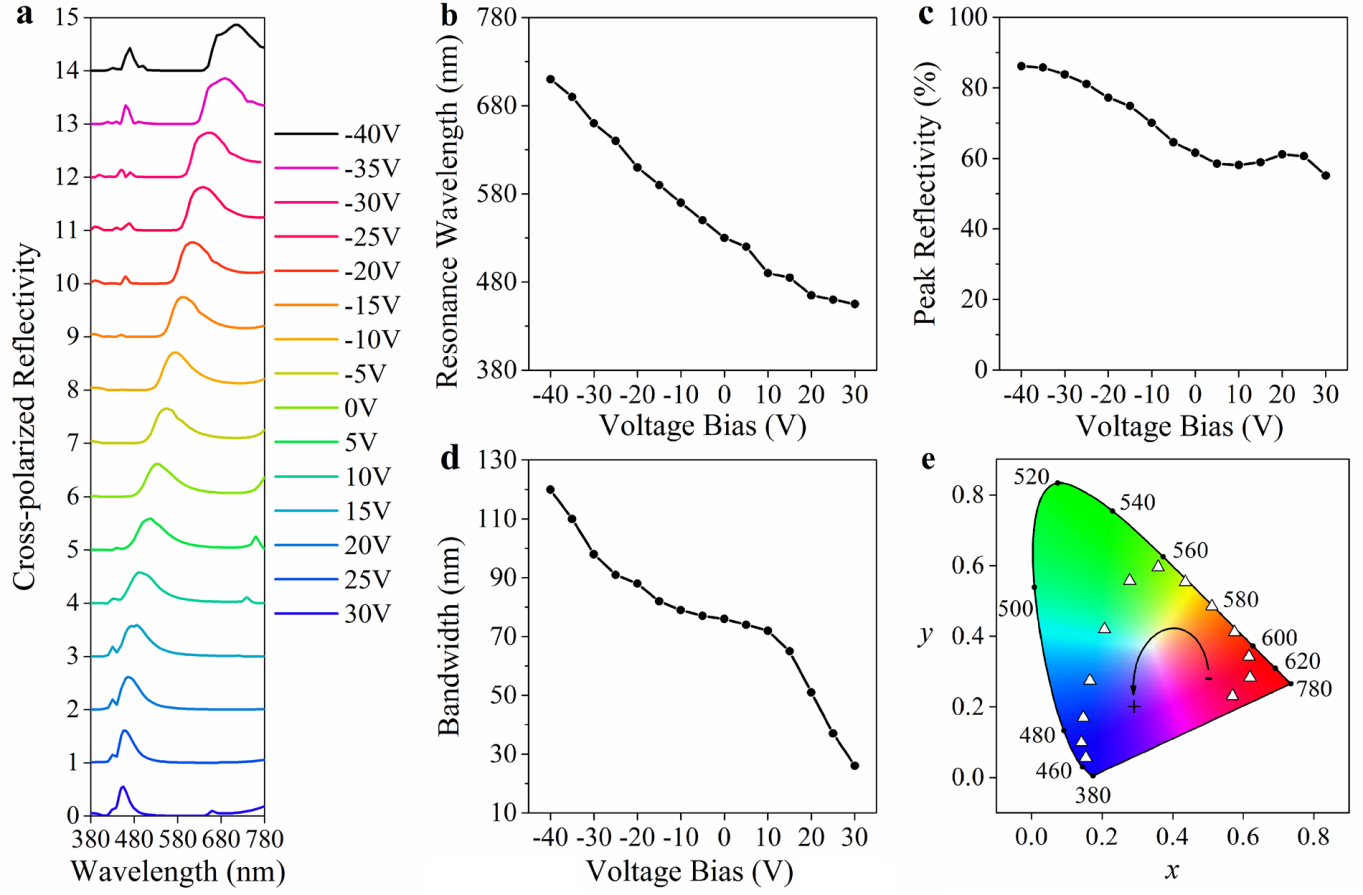
Colour selectivity by voltage modulation. (**a**) Cross-polarized reflection spectra tuned by voltage across the EO material. (**b**~**c**) are the resonance wavelength, peak reflectivity and bandwidth in (**a**), respectively. (**e**) CIE 1931 colour space with colour coordinate pairs derived from (**a**) at −30V to 30 V. The boundary curve of the colour space denotes the wavelength in nanometer scale. The triangle points arranged in anti-clockwise direction indicate colour coordinates achieved by our plasmonic nanostructure with a voltage range of −30V to 30 V. Spectra at −40V and −35V are not suitable for colour filter due to the secondary reflection peak so their coordinates are removed in the colour space.

Then at different voltage bias, the peak intensity of the optimized unit cell shifts in Fig. 7a. As voltage is tuned from positive bias to negative one, the refractive index of the EO material increased linearly^26^, followed by a continuous red shift in the reflection spectra. Once the voltage is larger than 35 V (not shown in Fig. 7a), blue wave is nearly absorbed in the cavity, and a wide-band reflectivity spectra covering red waveband appears. On contrary, when the voltage is lower than −35 V, a secondary reflection peak appears in the blue region influencing the colour purity, not suitable for colour filter. When the voltage bias is altered from −40 V to 30 V, the peak wavelength is gradually shifted from 710 nm to 455 nm in Fig. 7b, along with a peak reflectivity decreasing from 86% to 55% in Fig. 7c. For bandwidth, it narrows from 120 nm to 26 nm as plotted in Fig. 7d. Due to the secondary reflection peak in the spectra of −40 V and −35 V, they are not suitable for color filter. We take the spectra at −30 V to 30 V and calculate their colour coordinates in CIE 1931 space. In this voltage range, the peak reflectivity is higher than 60% and the bandwidth is narrower than 98 nm. As shown in Fig. 7e, with increasing voltage, the reflection colour varies from pink (−30 V at the bottom-right corner) to green (0 V at the top-middle corner), and finally to deep violet (30 V at the bottom-left corner). According to the color space, we can choose three points as red, green and blue color primary or more points to realize a multi-primary system.

**Figure 8:**
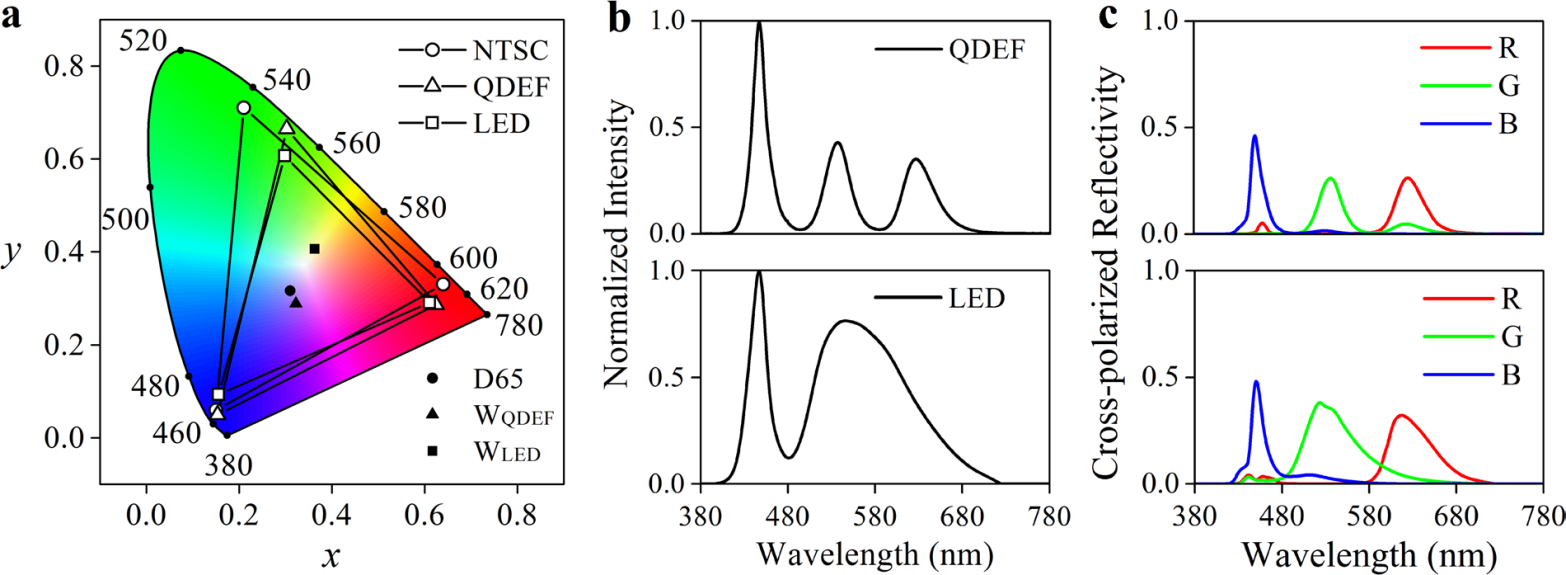
Gamut of the designed plasmonic display. (**a**) CIE 1931 colour space with colour coordinate pairs derived from reflection spectra of pixels multiply with white backlit. (**b**) Normalized spectra of QDEF and LED backlit used in (**a**). (**c**) Actual reflective spectra of RGB primary pixel assisted with backlit: top one with QDEF and bottom one with LED.

For a color display, operation of RGB colour filters is based on separation of a white backlit with the assistance of engineered plasmonic resonance. Taking typical white QDEF and LED backlit for examples, the RGB colour coordinates in Fig. 8a are derived from the actual display spectra (Fig. 8c), which are the product of the light source spectra in Fig. 8b and the RGB spectra in Fig. 7a. For a QDEF backlit, we choose −25 V for red, 0 V for green and 20 V for blue to maximize the gamut area, whose color coordinates are (0.624, 0.287), (0.302, 0.665) and (0.153, 0.049). The gamut of the chosen three primaries occupies 80% area of the National Television Systems Committee (NTSC) standard. In contrast, as for a LED backlit in Fig. 8a, the voltage chosen for red, green and blue color primary is −25V, 5 V and 25 V respectively, whose corresponding color coordinates are (0.610,0.291), (0.298.0.606) and (0.155,0.093). The gamut area with the LED backlit is 65% of the NTSC. The white points are calculated by mixing RGB pixel spectra with equal percentage. Although the white points deviate from D65, a comprehension method can modify it. In Fig. 8c, the peak wavelength of blue is 450 nm both for QDEF and LED. But that of green and red are different for two backlights: 535 nm and 625 nm for QDEF, while 525 nm and 615 nm for LED. Additionally, the bandwidth of blue is 15 nm both for QDEF and LED, whereas the bandwidth of green and red for QDEF is 30 nm and 37 nm respectively, narrower than that for LED (62 nm for green and 56 nm for red). The difference in spectra is the reason for the wider gamut of QDEF than LED.

### PWM-dependent brightness

**Figure 9:**
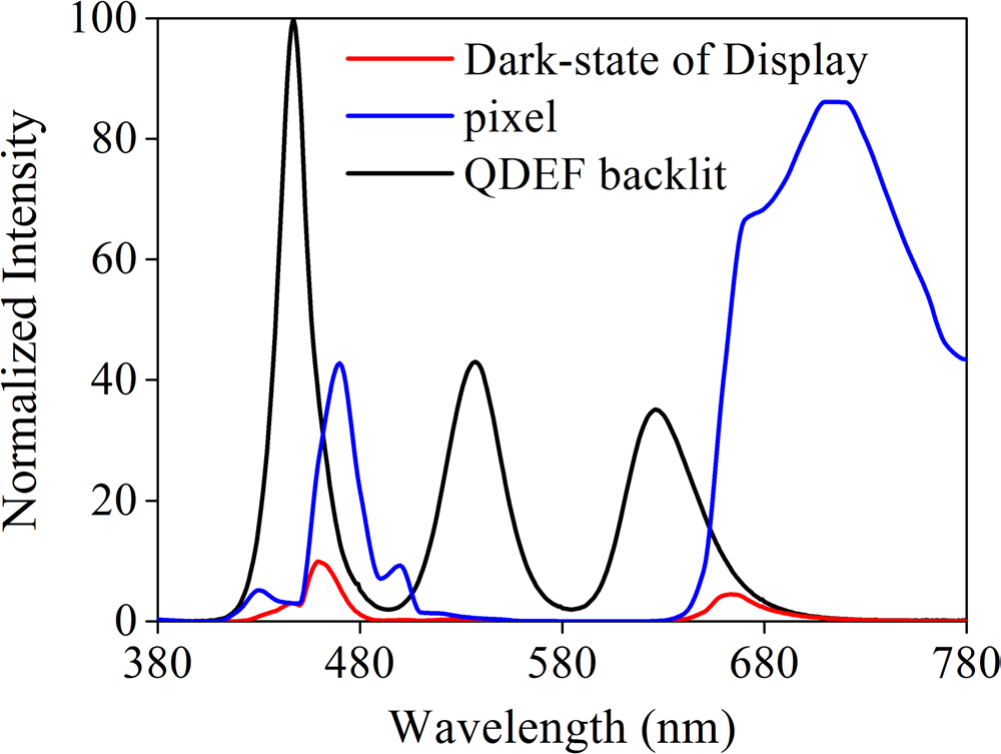
Reflection spectra for dark state. The red, blue and black line is for the dark-state spectra of the display, individual plasmonic pixel at −40V and QDEF backlit.

Besides full-colour tunability, a novel brightness control mechanism is adopted. As shown in Fig. 9, when tuning the resonance wavelength of the pixel to deep red region at −40 V, the actual reflectivity declines to lower than 9.9% because of the dips of backlit spectra. Although higher voltage may shift the resonance wavelength to infrared band, more secondary peaks would appeal in the blue band, leading to a more serious color crosstalk and higher reflectivity. Hence spectra at −40 V is denoted as a dark state. The brightness can be determined by tuning the duty cycle of bright and dark state. The maximum contrast ratio of R, G and B can be calculated by

2 $${\rm{CR}}=\frac{{\int }_{380}^{780}{R}_{R,G,B}(\lambda )\cdot {I}_{0}(\lambda )\cdot V(\lambda )d\lambda }{{\int }_{380}^{780}{R}_{{\rm{D}}}(\lambda )\cdot {I}_{0}(\lambda )\cdot V(\lambda )d\lambda },$$

where *I*_0_ is the backlit spectra, *V* is the relative spectral sensitivity of the human visual system in photopic condition, *R*_R,G,B,D_ are the spectra of red, green, blue and dark state, respectively, whose corresponding voltage is −25V, 0 V, 20 V and −40 V with QDEF backlit. The calculated CR is 10.63, 26.11 and 2.97 for R, G and B. We believe the proposed design can be improved by using a more proper white backlit or transferring to a transmission type and being assisted by liquid crystal.

In summary, we have theoretically demonstrated an active plasmonic display operating in the visible spectra with white backlit. Full color can be electrically modulated by utilizing a buffer layer and an EO material layer into a GSP-based metasuface. For a three primary system, the reflectivity is higher than 60%. Colour can be generated by applying a PWM voltage to mix the three primaries in a time sequence. Brightness can be tuned by the duty cycle of bright and dark state. When using a QDEF white backlit, the gamut of our design overlaps 80% area of NTSC colour space. The contrast ratio for red, green and blue is 10.63, 26.11 and 2.97, which can be improved by optimizing the backlit further. The presented concept and device have potentials for wearable optical applications and high-resolution displays.

## Method

The theoretical calculations are performed on one unit cell with periodic boundary conditions using FEM. The meshing size is lower than 20 nm, even finer to 10 nm in and around the antenna domain. Briefly, the air layer, antenna, buffer layer, ITO film, EO modulation layer, metal reflection layer and substrate are arranged along the *z*-axis. A 1um-thick air layer is considered in the simulations to avoid the effect of the local field enhancement on efficiencies. An *x*-polarized incident wave propagates along the *z*-axis from air with a spectra range from 380 nm to 780 nm. The boundary conditions for the *x*- or *y*-directions are symmetric, accounting for the periodicity of the model. Material properties of silver are adopted from experimental values by Johnson^32^. The refractive index of the ITO film can be retrieved from the square root of the permittivity *ε*, which is determined by the Drude model^33^

3 $$\varepsilon ={\varepsilon }_{\infty }-\frac{{{\omega }_{p}}^{2}}{{\omega }^{2}+i{\rm{\Gamma }}\omega },$$

where *ε*_∞_ is permittivity at infinite frequency, *ω* is angular frequency, *ω*_*p*_ is plasma frequency, and Γ is relaxation frequency. The corresponding value of *ε*_∞_, *ω*_*p*_ and Γ is 4.55, 2.0968 × 10^15^ rad/s and 724.6 THz. DAST is an EO organic crystal with a linear refractive index about voltage. The refractive index of DAST is given by *n* = *n*_0_ + (*dn*/*dE*) ∙ (*U*/*h*_EO_), where *n*_0_ = 2.2, *dn*/*dE* = 3.41 nm/V and *U* is the applied voltage. Its responding frequency is 18GHz^26,34,35^, fast enough for display demand. Both the ITO film and EO dielectric layer are assumed as homogeneous materials in the simulation. In the static electric simulation, the ITO film assumed as an equipotential volume is connected to a voltage source, while the metal layer is grounded. The relative static permittivity of ITO, DAST and silver is 9.3, 5.2 and 1, respectively. We firstly proceed the static electric simulation to get the refractive index of the EO material and then perform the electromagnetic field calculation. When the voltage changes from −40 V to 30 V, the refractive index of the EO material shifts from 3.109 to 1.518. The maximal electric field is 40 V/150 nm ≈ 0.267 kV/μm, which is acceptable according to the references^35,36^.

### Data availability

The datasets generated and analyzed during the current study are available from the corresponding author on reasonable request.
